# Dach1 transcription factor regulates the expression of peripheral node addressin and lymphocyte trafficking in lymph nodes

**DOI:** 10.1016/j.crimmu.2022.08.008

**Published:** 2022-08-21

**Authors:** Arisa Shintani, Shoko Fukai, Reika Nobusawa, Kanako Taniguchi, Tomohiro Hatatani, Hayato Nagai, Tomohiro Sakai, Takuji Yoshimura, Masayuki Miyasaka, Haruko Hayasaka

**Affiliations:** a Faculty of Science & Engineering, Department of Science, Graduate School of Science and Engineering, Kindai University, 3-4-1, Kowakae, Higashiosaka, Osaka, Japan; b WPI Immunology Frontier Research Center, Osaka University, 2-2, Yamada-oka, Suita, Osaka, Japan; c The Department of Pediatrics, Nara Medical University, 840 Shijo-cho, Kashihara, Nara, Japan

**Keywords:** Lymphocyte, Lymph node, Endothelial cell, Transcription factor, Trafficking, HEV, High endothelial venules, ECs, endothelial cells, LNs, Lymph node, PPs, Peyer's patches, ILNs, Inguinal lymph nodes, MLNs, Mesenteric lymph nodes, PNAd, peripheral node addressin, MAdCAM-1, mucosal addressin cell adhesion molecule-1, mAb, monoclonal antibody

## Abstract

Lymphocytes regulate the immune response by circulating between the vascular and lymphatic systems. High endothelial venules, HEVs, special blood vessels expressing selective adhesion molecules, such as PNAd and MAdCAM-1, mediate naïve lymphocyte migration from the vasculature into the lymph nodes and Peyer's patches. We have identified that DACH1 is abundantly expressed in developing HEV-type endothelial cells. DACH1 showed a restricted expression pattern in lymph node blood vessels during the late fetal and early neonatal periods, corresponding to HEV development. The proportion of MAdCAM-1^+^ and CD34^+^ endothelial cells is reduced in the lymph nodes of neonatal conventional and vascular-specific Dach1-deficient mice. Dach1-deficient lymph nodes in adult mice demonstrated a lower proportion of PNAd^+^ cells and lower recruitment of intravenously administered lymphocytes from GFP transgenic mice. These findings suggest that DACH1 promotes the expression of HEV-selective adhesion molecules and mediates lymphocyte trafficking across HEVs into lymph nodes.

## Highlights

• The high endothelial venules, HEVs, develop in a tissue-specific manner and permit lymphocyte trafficking.
• The transcription factor DACH1 exhibit a restricted expression pattern in the blood vessels of developing lymph nodes.
• The blood vessel-specific *Dach1*-deficient lymph nodes exhibit a reduced proportion of HEVs and lymphocyte recruitment.

## Graphical abstract

**Figure:**
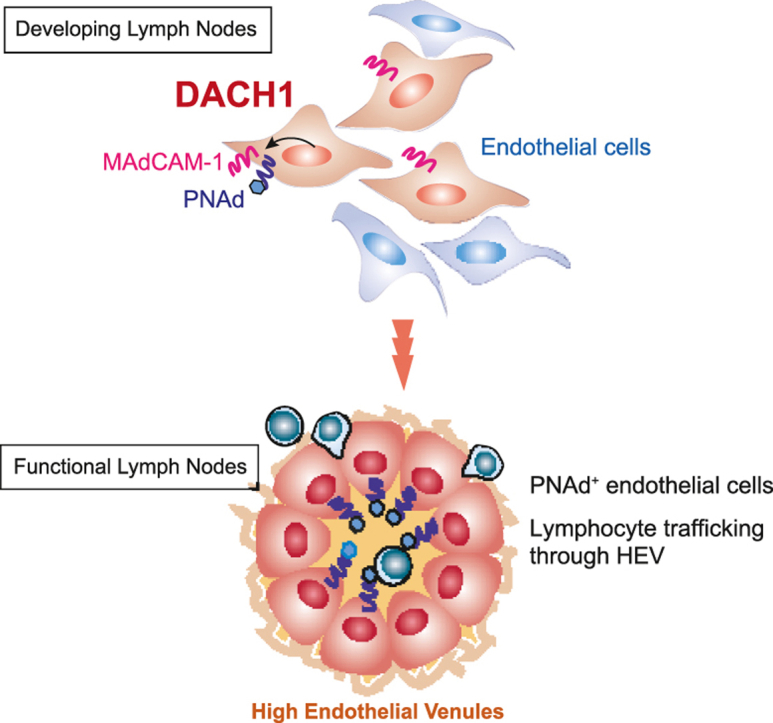

## 1 Introduction

By migrating from blood to secondary lymphoid tissue and monitoring pathogen invasion, lymphocytes contribute to an efficient immune response. Lymphocytes migrate from the blood into lymph nodes (LNs) and Peyer's patches (PPs) via specialized high endothelial venules (HEVs), which are distinguished from other blood vessels by their tall and thickened endothelial cells (ECs) and a distinct morphological feature that includes a thick basement membrane composed of extracellular matrix (Miyasaka and Tanaka, 2004; Hayasaka et al., 2010; Umemoto et al., 2011). HEV endothelial cells (HEV-ECs) express a group of adhesion molecules crucial for the selective trafficking of naïve lymphocytes from blood to lymphoid tissues. PNAd (peripheral node addressin) is composed of sulfated and glycosylated molecules with sialyl-lewis X sugar chain structure attached to core proteins, including GlyCAM-1, CD34, nepmucin, endomucin, and MAdCAM-1 (mucosal addressin cell adhesion molecule-1) (Lasky et al., 1992; Baumheter et al., 1993; Kanda et al., 2004; Umemoto et al., 2006; Berg et al., 1993). The interaction between these sialomucins and L-selectins expressed on lymphocytes causes weak adhesion between HEV-ECs and lymphocytes, resulting in lymphocyte rolling on the HEV-ECs. MAdCAM-1 is selectively expressed on HEV-ECs in mucosal lymphoid tissues. Next, chemokines such as CCL21, CCL19, CXCL10, CXCL12, and CXCL13, expressed and attached to HEV-ECs, activate lymphocyte integrins under shear forces during lymphocyte rolling on HEV-ECs. Finally, the interaction of activated integrins with immunoglobulin superfamily adhesion molecules promotes lymphocyte migration across HEVs.

At the late embryonic stage during LN development, some blood vessels exhibit the morphological characteristics of HEV with a thick basement membrane (Hayasaka et al., 2010), and lymphocyte accumulation begins 1–2 days after birth (Cupedo et al., 2004). At this perinatal stage, all HEV-ECs express MAdCAM-1 in both peripheral and mucosal LNs, implying that MAdCAM-1 plays a role in the recruitment of lymphoid tissue inducer (LTi) cells expressing α_4_β_7_ integrin to LNs (Mebius et al., 1996). Conversely, MAdCAM-1 is not a selective marker molecule for HEV-ECs in the fetal period due to its universal expression pattern in all veins, which lack the HEV morphological phenotypes (Hashi et al., 2001). Our previous study demonstrated that MAdCAM-1 expression in LN blood vessels is divided into either MAdCAM-1^+^ or MAdCAM-1^-^ during the late embryonic period (Hayasaka et al., 2010), suggesting that functional HEVs is formed at a specific point between the late embryonic and the neonatal periods. While MAdCAM-1 expression is predominant in both mucosal and peripheral LNs at birth, it gradually shifts to PNAd 24 h after birth and almost completely disappears by 3-weeks after birth (Mebius et al., 1996). In contrast, MAdCAM-1 expression in mucosal LNs does not completely disappear; therefore, HEVs of MAdCAM-1^+^ and PNAd^+^ are detected in mesenteric lymph nodes (MLNs) and PPs.

Our previous DNA microarray analysis of newborn mouse HEV-ECs revealed that they preferentially express many angiogenesis-associated genes previously expressed in endothelial progenitor and immature ECs (Hayasaka et al., 2010). This finding allows us to hypothesize that specific transcription factors are expressed and induce HEVs from immature blood vessels from the late embryonic stage to the neonatal period. In this study, we looked for transcription factors involved in HEV-EC differentiation. We discovered *Dach1*, which is only expressed during early HEV development.

*Dach1* is a mouse homologue gene of Dachshund, Dac, an essential gene for retinal and limb bud formation in Drosophila (Mardon et al., 1994). At the developmental stage, mouse *Dach1* is expressed in the central nervous system, neural crest, and limb buds, and *Dach1* -deficient mice die shortly after birth due to feeding disorders and cyanosis (Backman et al., 2003; Davis et al., 2001). These findings suggest that *Dach1* is essential for the central nervous system and lung function. However, the precise roles of DACH1 in these organs are unknown. In recent years, researchers reported physiological DACH1 functions using tissue-specific *Dach1*-deficient and *Dach1* overexpressing mice, where DACH1 is highly expressed in developing coronary arteries and regulates coronary angioplasty (Chang et al., 2017; Raftrey et al., 2021). In a pathological context, DACH1 acts as a transcriptional repressor in kidney podocytes and tubule cells, and it is associated with the development of chronic kidney disease (Doke et al., 2021; Cao et al., 2021). DACH1 functions as a tumor suppressor in various cancer types by binding to multiple gene promoters and acting as a transcription repressor (Wu et al., 2011; Chen et al., 2015; Xu et al., 2017; Watanabe et al., 2011; Yu et al., 2019).

This study examined *Dach1*-deficient LNs to determine how DACH1 affects HEV formation *in vivo*. We demonstrated that the blood vessel EC-specific *Dach1*-deficiency hampered PNAd expression and lymphocyte trafficking into peripheral LNs, implying that DACH1 expression is required for adhesion molecule expression and efficient lymphocyte trafficking across HEVs into LNs.

## 2 Material and methods

### 2.1 Animals

C57BL/6J and GFP transgenic mice (CAG-EGFP) were purchased from Nihon SLC (Hamamatsu, Japan). NOD/ShiJcl mice were purchased from CLEA Japan, Inc. (Tokyo, Japan). Conventional *Dach1* null mice with the exon 1 deletion (Backman et al., 2003) were generated by transplanting frozen CBB6-Dach1 mouse embryos (obtained from the European Mouse Mutant Archive) into surrogate mother followed by sibling mating of heterozygous mice. A *Dach1* mutant mice (RBRC10097, RIKEN BioResource Research Center) were generated from an embryonic stem cell clone with *Dach1* gene floxed by loxP sequences on each side of the first exon. The vascular endothelium-specific *Dach1*-deficient mice (Dach1-cKO) were obtained by breeding between the female mice for the homozygous floxed *Dach1* allele (fl/fl) and the male *Dach1* fl/fl mice carrying the Tie2-Cre allele derived from the B6.CG-Tg (Tek-Cre) 1Ywa mice (Kisanuki et al., 2001) (Tie2-Cre). The floxed allele of the offspring was determined by PCR using KOD FX Neo (TOYOBO) and a pair of PCR primers 5′- gag gtg act gtg gcc gcg gct ga -3′ and 5 ′- tgc gct cgc tct ttc tta acc tc -3'.

Tissues were harvested from mice given a lethal dose of isoflurane via inhalation. The experimental protocols for use of laboratory animals were approved by the Ethics Review Committee of Osaka University Graduate School of Medicine and that of Kindai University.

### 2.2 Microarray and quantitative RT-PCR analysis

MLNs were harvested from newborn or four-day-old C57BL/6 mice and treated with 0.5 mg/ml type I collagenase in RPMI-1640 containing 10% FCS for 45 min at 37 °C. The partially dissociated cells were slowly passed through a 22-gauge needle a few times. After a brief centrifugation, the cell pellet was resuspended in 0.2% trypsin/0.5 mM EDTA in PBS and incubated for 15 min at 37 °C. Digestion was stopped by adding FCS to a final concentration of 10%, and the cells were slowly passed through a 22-gauge needle a few times, followed by filtration through 0.1 mm nylon mesh to obtain a single cell suspension. The single cell suspension was treated with anti-mouse CD16/CD32 monoclonal antibody (mAb), and then stained with FITC-conjugated anti-CD45 mAb, APC-conjugated anti-CD34 mAb and biotin-anti-MAdCAM-1 mAb. After being washed with BSA/HBSS containing 1 mM EDTA, the cells were stained with streptavidin-PE and 7-AAD. After amplifying the fluorescence intensity of PE using FASE kit-PE (Miltenyi Biotec), the CD34^+^MAdCAM-1^+^ and CD34^+^MAdCAM-1^-^ type cells gated on the CD45^−^ and 7-AAD^-^ stromal population were sorted by FACSAria.

Total RNA was extracted from using RNAqueous ®-4PCR kit (Ambion). RNA (5 ng) was subjected to the first strand cDNA synthesis, amplification, fragmentation and biotin-labeling by the Ovation Biotin System (NuGEN). The fragmented biotinylated cDNA was added to the GeneChip Mouse Genome 430 2.0 Array (Affymetrix), hybridized overnight, scanned using the 428 Affymetrix Scanner (Affymetrix). Trimmed average signal (500) of the array was obtained by finding the mean of 96% of the probe sets (2% of highest and lowest values were excluded) and was used as a reference for normalization. The data were then analyzed by GeneSpring Bioinformatics software (Agilent). The analyses were performed twice, using pooled RNA from 84 to 74 newborn MLNs, respectively.

For quantitative RT-PCR, 94 four-week-old MLNs were collected. The pooled RNA sample was subjected to the first strand cDNA synthesis and amplification by WT OVATION™ Pico RNA Amplification System (NuGEN). Real-time quantitative RT-PCR was conducted with SYBR Green PCR master mix (Applied Biosystems) on the ABI 7900 FAST instrument. The 2(-Delta Delta C(T)) analysis was used to calculate the relative amount of expression of individual genes in relation to *gapdh* control. The primer sequences used are described below.

Dach1 forward: 5′-ccaaagtggcttcctttacgg-3′, Dach1 reverse: 5′-atctccaaccgcttcagcttg-3′

Tal1 forward: 5′-gcctaagctgcagttccctaca-3′, Tal1 reverse: 5′-ttacggacccaatggacttcc-3′

Fli1 forward: 5′-tcctaccatgcccatcaacag-3′, Fli1 reverse: 5′-gaggtgacaggcatggaggat-3′

Irf8 forward: 5′-gaggtctttcgacagttcaaatca-3′, Irf8 reverse: 5′-ccgcagaagggcaaagg-3′

Nr5a2 forward: 5′-aactgctggagtgagctcttgat-3′, Nr5a2 reverse: 5′-ttcccatgcgccacttgt-3′

### 2.3 Immunohistochemistry

Serial sections of fresh-frozen specimens were subjected to immunohistochemical staining. Frozen tissue sections were incubated with the antibodies and fluorescent reagents listed on Table S1. For detection of DACH1 and CCL21, a tyramide signal amplification system (Thermo Fisher Scientific) was used with horseradish peroxidase (HRP)-conjugated goat anti-rabbit IgG (2.5 μg/ml), HRP-conjugated streptavidin and Alexa Fluor 568 or Alexa Fluor 488 tyramide. For whole-mount immunofluorescence, popliteal lymph nodes (pLN) were treated with 4% paraformaldehyde and with PBS containing 4% glycine for 30 min at 4 °C. After dehydration in methanol, the tissues were incubated with 10% Immunoblock (DS Pharma) in PBS containing 0.1% TritonX-100 for 1 h at room temperature. The tissues were stained with biotinylated MECA-79 mAb for 1 week at 4 °C, subsequently with Alexa Fluor 568-labeled streptavidin for 3 days. The immunohistochemical stained images were captured by confocal microscopy (FV1000-D, Olympus) and were analyzed using ImageJ software (NIH).

### 2.4 Immunization using CFA/OVA

A 100 μl 1:1 mixture of complete Freund's adjuvant (CFA; Sigma) and ovalbumin (1 mg/ml) were injected into 3 locations on the back of 8-week-old female C57BL/6 mice. The brachial LNs and the axillary LNs were collected 4 days after injection.

### 2.5 Adoptive transfer

Splenocytes (1 × 10^6^) from GFP transgenic mice were isolated and injected into the tail vein of *Dach1* fl/fl mice and male Tie2-Cre^+^ *Dach1* fl/fl mice. Single-cell suspensions from spleen, inguinal lymph nodes (ILN), and MLNs were prepared from the recipient mice 1 h after transfer. The percentage of GFP^+^ cells in each tissue was determined by flow-cytometric analysis using a FACS Aria ΙΙ (BD Biosciences) and the FlowJo software (BD Biosciences). The transferred GFP^+^ cells in ILNs or MLNs were standardized by the number of GFP^+^ splenocytes in the same individual. For whole-mount analyses of LNs, mice were intravenously injected with GFP^+^ splenic cells (1.5 × 10^7^ cells/mouse). One hour after cell administration, mice were injected with 4 μg of eFluor660-labeled CD34 mAb and sacrificed 15 min later, and ILNs and pLNs were harvested. GFP^+^ lymphocytes and CD34^+^ blood vessels were detected by confocal microscopy. For pLNs, Z-plane images of the eFluor660 dye were acquired every 5 μm (approximately 150 μm thickness) and were analyzed by Metamorph software (Molecular Devices Company).

### 2.6 Flow cytometry

The tissues were treated with RPMI-1640 (Wako) containing 0.1% BSA, 1 mg/ml Collagenase type Ι (Wako), and 2 μg/ml DNase Ι (Sigma) for 45 min at 37 °C, and the cell suspension was overlaid on 40% FCS (fetal calf serum; PAA Laboratories) for 1 h. The lower stroma cell fraction was collected and treated with 0.2% trypsin and 0.5 mM EDTA (Wako) for 15 min at 37 °C, subsequently with 10 μg/ml DNase Ι and 10% FCS. A single cell suspension passed through a nylon mesh with 108 μm pore size were then treated with culture supernatant containing anti-CD16/32 antibody, followed by FITC-labeled anti-CD45, biotin-labeled MECA-79, biotin-labeled MECA-89, and eFluor660-labeled CD34 mAbs, PE-labeled streptavidin, and 7-aminoactinomycin D (7-AAD). The data analysis and interpretation were carried out using a FACS Aria ΙΙ and the FlowJo software.

## 3 Results

### 3.1 Transcription factors selectively expressed in neonatal HEV ECs

In a previous DNA microarray analysis of HEV-ECs isolated from newborn MLNs (Hayasaka et al., 2010), we found that 16 transcription factors that are more than 8-fold more abundant in neonatal CD34^+^MAdCAM-1^+^ ECs (HEV-type ECs) than CD34^+^MAdCAM-1^-^ ECs (non-HEV-type ECs). To identify transcription factors that are highly expressed in four-day-old HEV-type cells, quantitative PCR was performed on each of the screened transcription factors, and five of 16 genes (*Tal1, Dach1, Fli1, Irf8, and Nr5a2)* were significantly over-expressed in HEV-type cells compared to non-HEV-type cells with a range of mean expression difference from 65 to 975 (Table 1). Our preliminary *in situ* hybridization analysis has shown undetectable expression of *Fli1, Irf8, and Nr5a2* and faint signals of *Dach1* and *Tal1* in newborn MLNs (data not shown). In addition, *Tal1* was broadly expressed and is known as a marker of the endothelial lineage and regulates vascular development (De Val and Black, 2009), whereas *Dach1* gene exhibits a limited tissue expression profile according to the mouse Gene Expression Database (GXD). Therefore, we focused on the *Dach1* gene for further study.

**Table 1:** **The expression of transcription factors up-regulated in perinatal HEV-type ECs**^a^. CD34^+^MAdCAM-1^+^ and CD34^+^MAdCAM-1^-^ cell fractions of newborn and 4-day-old C57BL/6 MLNs were obtained and subjected to microarray analysis and quantitative RT-PCR analysis, respectively. The mean signal intensity of two measurements was used for normalization by a housekeeping gene (*gapdh*). A fold change defined as the ratio of expression levels in CD34^+^MAdCAM-1^+^ (HEV-type) over CD34^+^MAdCAM-1^-^ (non-HEV-type) cells.

	Microarray			qRT-PCR
	Signal intensity relative to *gapdh*		Fold change HEV/non-HEV	Fold change HEV/non-HEV
Transcription Factors	HEV-type cells	Non HEV-type cells		
*Tal1*	0.54	0.014	37	975
*Dach1*	0.23	0.009	25	179
*Fli1*	1.09	0.06	18	382
*Irf8*	0.17	0.0085	20	65
*Nr5a2*	0.62	0.027	23	315

a The data were extracted from the same database used in Reference 2.

### 3.2 Analysis of DACH1 expression in mouse secondary lymphoid tissue

**Fig. 1:**
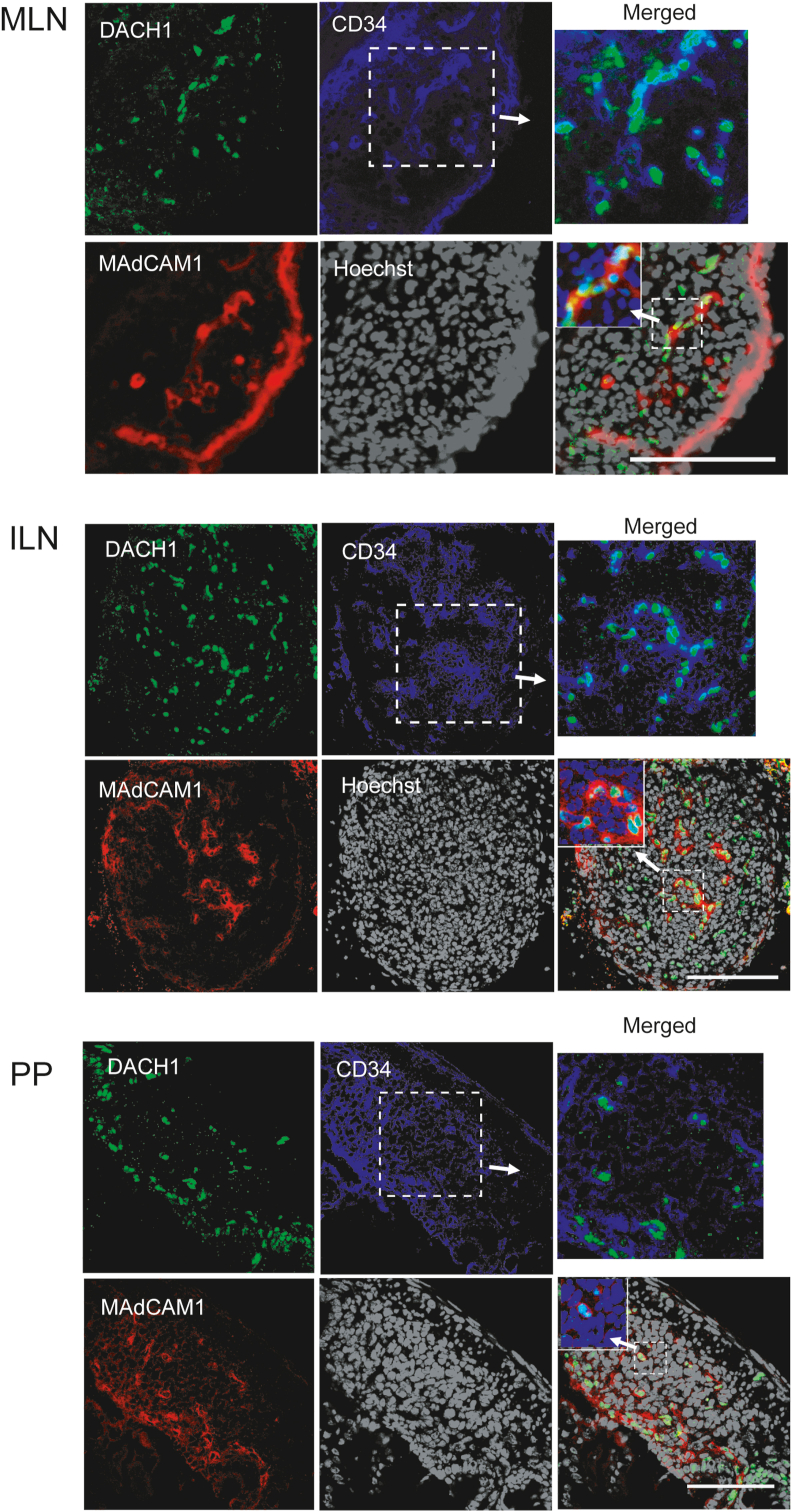
**The expression of DACH1 in the newborn mouse secondary lymphoid tissues.** Mesenteric lymph nodes (MLNs), inguinal lymph nodes (ILN), Peyer's patches (PP) were harvested from newborn C57BL/6 mice. Immunohistochemistry was used to detect DACH1 (green, top left), MAdCAM-1 (red, bottom left), and CD34 (blue, top center) expression. Nuclei were stained with Hoechst33342 (bottom center). Top right: merged images of DACH1 and CD34. Bottom right: merged images of DACH1, CD34 and MAdCAM-1. Inset: merged images of DACH1, MAdCAM-1 and Hoechst33342. Data are representative of 7 experimental repeats with 7 mice in each group. Scale bar: 100 μm. (For interpretation of the references to colour in this figure legend, the reader is referred to the Web version of this article.)

To investigate the involvement of DACH1 in HEV development, we investigated its expression in neonatal mouse secondary lymphoid tissues. DACH1 was found primarily in the nucleus of MAdCAM-1^+^CD34^+^ vascular-like structures and marginally in MAdCAM-1^−^CD34^+^ cells in MLNs and inguinal LNs (ILNs), but not in the MAdCAM-1^+^ subcapsular sinus below the capsule, as shown in Fig. 1. DACH1 expression was detected as dot-shaped signals in PPs in MAdCAM-1^+^CD34^+^ cells and MAdCAM-1^−^CD34^−^ non-ECs. DACH1 expression was undetectable in the spleen, which is consistent with the absence of HEVs in this organ (Fig. S1). These results indicate that DACH1 is highly expressed in neonatal HEV-ECs in MLNs and peripheral LNs.

**Fig. 2:**
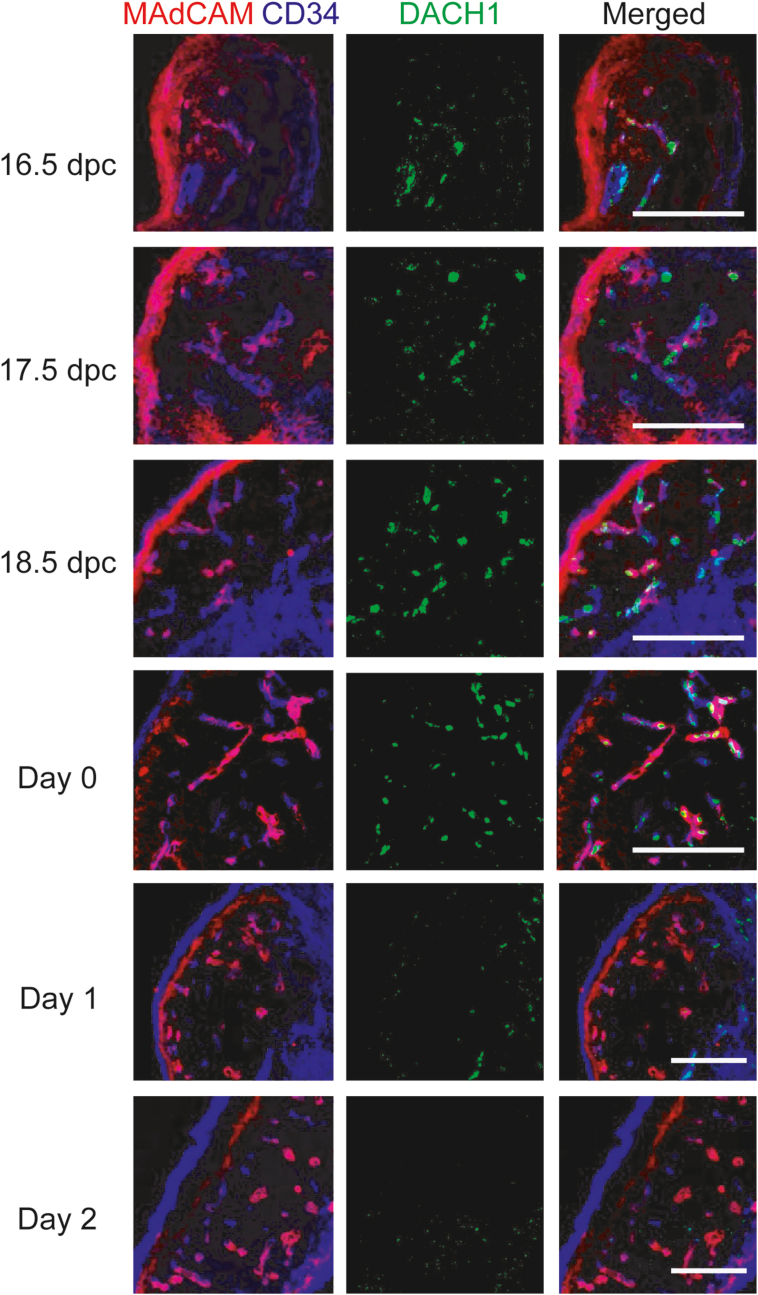
**Time course of DACH1 expression pattern in fetal and postnatal MLNs.** MLNs were collected from 16.5 dpc to two days after birth, and DACH1, MAdCAM-1, and CD34 expression were analyzed by immunohistochemistry. Scale bar: 100 μm. Data are representative of 3 experimental repeats with 3 mice in each group.

Immunohistochemical staining was performed on late fetal to postnatal MLNs to investigate DACH1 expression during the developmental stage. DACH1 expression was observed within the nucleus of MAdCAM-1^+^CD34^+^ HEV-ECs from E16.5 to day 0, as shown in Fig. 2, but was undetectable by day 2. At all stages of development, no evidence of DACH1 expression in the subcapsular sinus or large blood vessels was found. According to these findings, DACH1 is primarily expressed in HEV-like ECs during the fetal to neonatal period, which corresponds to HEV-EC development., supporting the idea that *Dach1* contributes to HEV-EC development.

### 3.3 DACH1 expression in inflamed tissues

It has been reported that PNAd^+^ MAdCAM-1^+^ HEV-like vascular structure develops ectopically in the pancreas of non-obese diabetes model NOD mice due to lymphocyte infiltration (Faveeuw et al., 1994). We sought to determine whether DACH1 expression is linked to HEV-like structures that appear ectopically in chronic inflammatory conditions. In the pancreas of 13-week-old NOD mice, there was clear lymphocyte infiltration and PNAd^+^ MAdCAM-1^+^ blood vessels inside the islets, but DACH1 expression was undetectable (Fig. S2A). This finding suggests that DACH1 expression is not induced in chronically inflamed HEV-like vascular ECs. Further, we examined whether DACH1 expression induces HEV endothelial cell proliferation after antigen administration. Compared to the control, LNs, the number of HEVs in the enlarged brachial and axillary LNs was increased four days after OVA immunization of 8-week-old mice, whereas DACH1 expression was not observed in the PNAd ^+^ HEVs (Fig. S2B). These results suggest that DACH1 is not involved in HEV remodeling during an immune response.

### 3.4 Expression of HEV-selective molecules in conventional *Dach1*-deficient mouse LNs

**Fig. 3:**
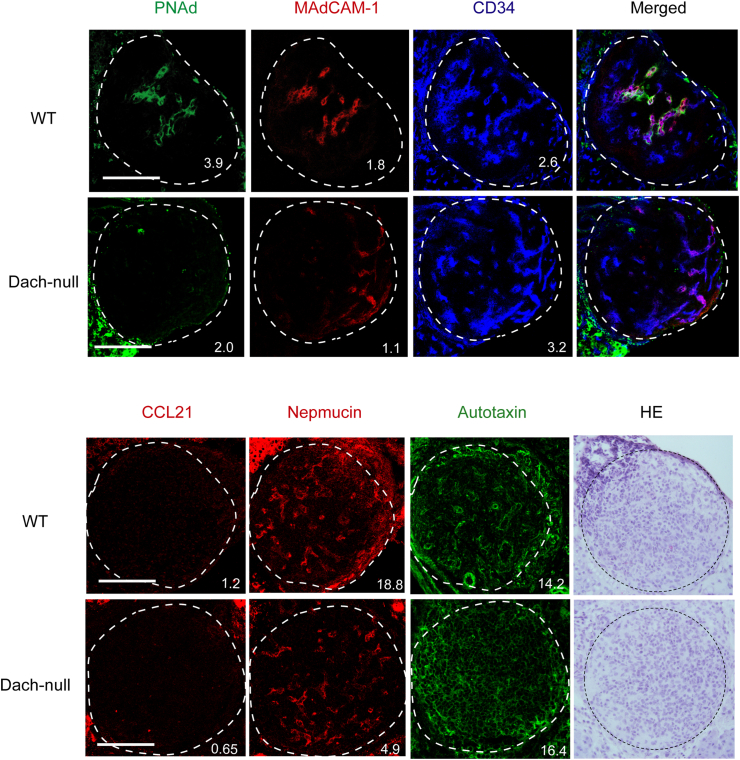
**The expression of HEV signature molecules in wild-type and the conventional *Dach1*-null peripheral LNs.** Newborn ILNs of *Dach1*-null and the wild-type littermate control were serially cryosectioned and analyzed by immunohistochemistry. The images were digitally processed with a median filter (2.0 pixel radius) using ImageJ software. The numbers represent percentage of the mean fluorescent intensity (bottom) per LN area. Scale bar: 100 μm. Data are representative of 2 experimental repeats with 2 littermate mice in each group.

We analyzed DACH1, MAdCAM-1, and CD34 expression in MLNs and ILNs of *Dach1*-null or wild-type control mice at late embryonic and neonatal stages, to determine whether it contributes to HEV formation. We confirmed that no obvious DACH1 expression observed in *Dach1*-null MLNs and ILNs, while it was clearly detected in a significant proportion of blood vessels in wild-type tissues (Fig. S3). Although MAdCAM-1^+^CD34^+^ structures were found in both Dach1-null and wild-type MLNs and ILNs, they appeared to be less abundant in *Dach1*-null neonatal MLNs and embryonic ILNs than the wild type. In addition, we examined the expression of HEV-selective genes such as PNAd, CCL21, nepmucin, and autotaxin in newborn *Dach1*-null ILNs. As shown in Fig. 3, PNAd expression in CD34^+^ blood vessels were significantly reduced (MFIs were 3.9 and 3.1 in WT, whereas 2.0 and 0.4 in *Dach1*-null in two independent littermate samples). MAdCAM-1 and nepmucin expression were also marginally reduced in *Dach1*-null ILNs. CCL21 chemokine expression was not detected in either the *Dach1*-null or wild-type control neonatal LNs, and autotaxin expression was at comparable levels. Based on these findings, we hypothesized that DACH1 expression influences the expression of HEV-selective molecules during HEV development.

### 3.5 Expression of HEV differentiation marker molecules in the blood vessel-specific *Dach1*-deficient mice

**Fig. 4:**
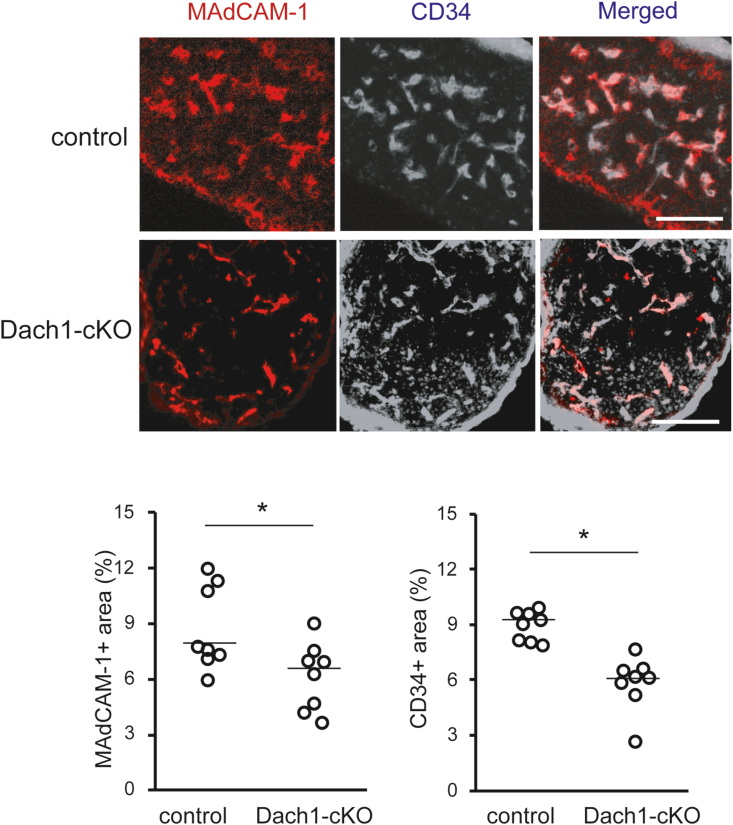
**Histological analysis of neonatal LNs in endothelial-specific Dach1-cKO mice.** The percentage of MAdCAM-1^+^ and CD34^+^ in each tissue section of neonatal MLNs were quantified by ImageJ software. Data are representative of 6 tissue sections from littermate mice. Each plot in the scatter plot represents a single tissue section's value, and each line represents the median. The Mann-Whitney *U* test was used to analyze the data. *, P < 0.05. NS, not significant. Scale bar: 100 μm.

**Fig. 5:**
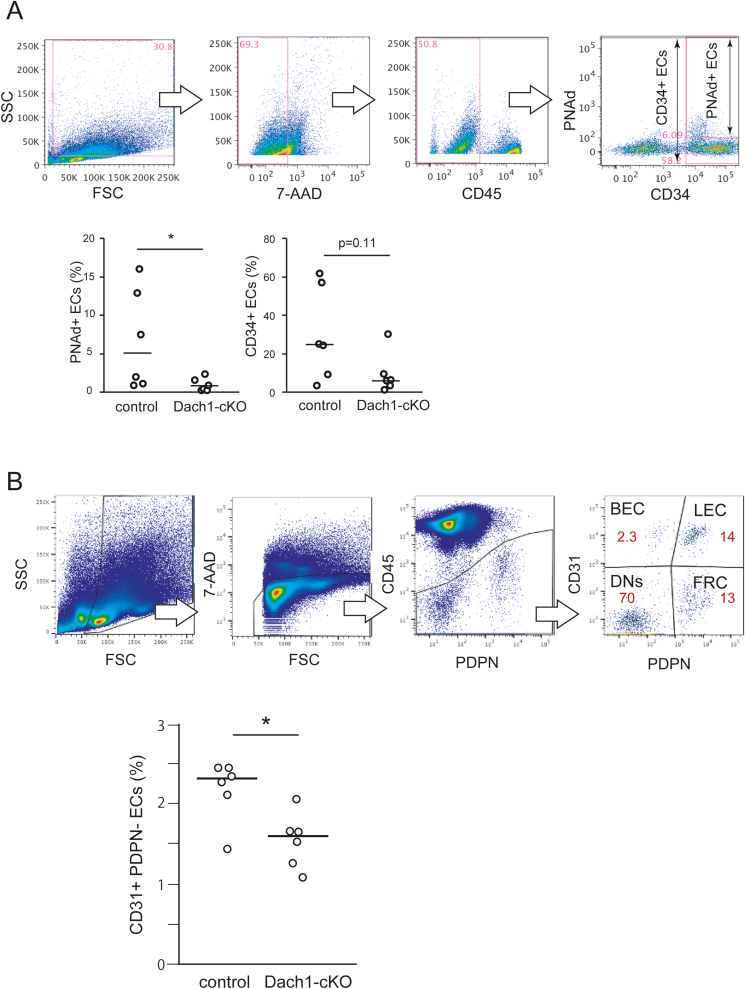
**Flow cytometric analysis of ECs in Dach1-cKO ILNs.** (A) The percentage of HEV-ECs in 3-week-old ILNs were quantified by flow cytometry. CD34^+^ and PNAd^+^ cells in 7-AAD^-^ CD45^-^ stromal cell populations were analyzed. In each experiment, samples were prepared from the bilateral ILNs of three littermate mice per group and pooled. Each plot shows the value of each pooled sample in 6 experimental repeats. Mann-Whitney's *U* test was used as the significance test. The lines show the median value. *, P < 0.05, NS: not significant. (B) The percentage of CD31^+^podoplanin^-^ blood endothelial cells (BEC), CD31^+^podoplanin^+^ lymphatic endothelial cells (LEC), CD31^-^podoplanin^+^ fibroblastic reticular cells (FRC) and CD31^-^podoplanin^-^ double negative (DN) subsets in CD45^-^ non-myeloid cells in the bilateral ILNs of each mouse. Results are representative of two experimental repeats with three mice per group in each repeat. The Mann-Whitney *U* test was used to analyze the data. *, P < 0.05.

As previously reported, the *Dach1*-null mice showed postnatal lethality (Backman et al., 2003). Therefore, we generated vascular endothelium-specific *Dach1* deficient mice (Dach1-cKO) to analyze the effect of *Dach1* deficiency on the expression level of HEV-EC differentiation markers in adult LNs (Fig. S4A). Immnohistochemical analysis showed an approximately 70% reduction in DACH1 expression in the Dach1-cKO MLNs (Fig. S4B). Microscopic observation revealed that the number of MAdCAM-1^+^ blood vessels in newborn Dach1-cKO MLNs was comparable to the control, whereas statistical analysis of tissue images revealed that the number of MAdCAM-1^+^ and CD34^+^ areas were significantly reduced in Dach1-cKO (Fig. 4). To assess the expression of HEV-selective molecules more precisely, we performed flow cytometry analysis on 3- and 6-week-old ILNs, measuring the percentage of PNAd^+^ and CD34^+^ cells in a stromal cell population after excluding 7-AAD^+^ dead cells and CD45^+^ myeloid cells (Fig. 5A). The proportion of PNAd^+^ cells in ILNs was significantly lower in 3-week-old Dach1-cKO (Fig. 5A) and a similar trend was observed in 6-week-old Dach1-cKO mice (Fig. S5A). CD34^+^ cells showed a trend toward reduction when compared to the control in ILNs (Fig. 5A), whereas a similar quantitative analysis of MLN showed no significant change in the proportion of PNAd^+^, MAdCAM-1^+^, and CD34^+^ cells (Fig. S5B). To further analyze the percentage of endothelial cells in LNs, we dissected CD45^-^ non-myeloid cells by CD31^+^podoplanin^-^ blood endothelial cells (BEC), CD31^+^podoplanin^+^ lymphatic endothelial cells (LEC), CD31^−^podoplanin^+^ fibroblastic reticular cells (FRC) and CD31^-^podoplanin^-^ double negative (DN) subsets (Fig. 5B). The proportion of BEC in ILNs was significantly lower in Dach1-cKO mice, suggesting that the lack of DACH1 reduces LN blood vessels. These results suggest that vascular EC-specific *Dach1* deficiency reduces the expression of HEV differentiation marker molecules and angiogenesis in LNs, although its effects differ between peripheral and mucosal LNs.

A visual observation of the vascular architecture in pLNs was comparable between 3-week-old control and Dach1-cKO (Fig. S6A). Although the whole mount immunostaining with anti-PNAd mAb did not provide sufficient signal levels for image analysis, large blood vessels with thickened endothelial cells in pLNs were clearly detected after intravenous anti-CD34 mAb administration (Fig. S6B). There was no statistical difference between control and Dach1-cKO in the percentage of positive signals, whereas the vessel length was significantly reduced in the Dach1-cKO, suggesting that *Dach1* deficiency affects the LN architecture (Fig. S6B).

### 3.6 Effects of Dach1-deficiency on lymphocyte trafficking through HEVs

**Fig. 6:**
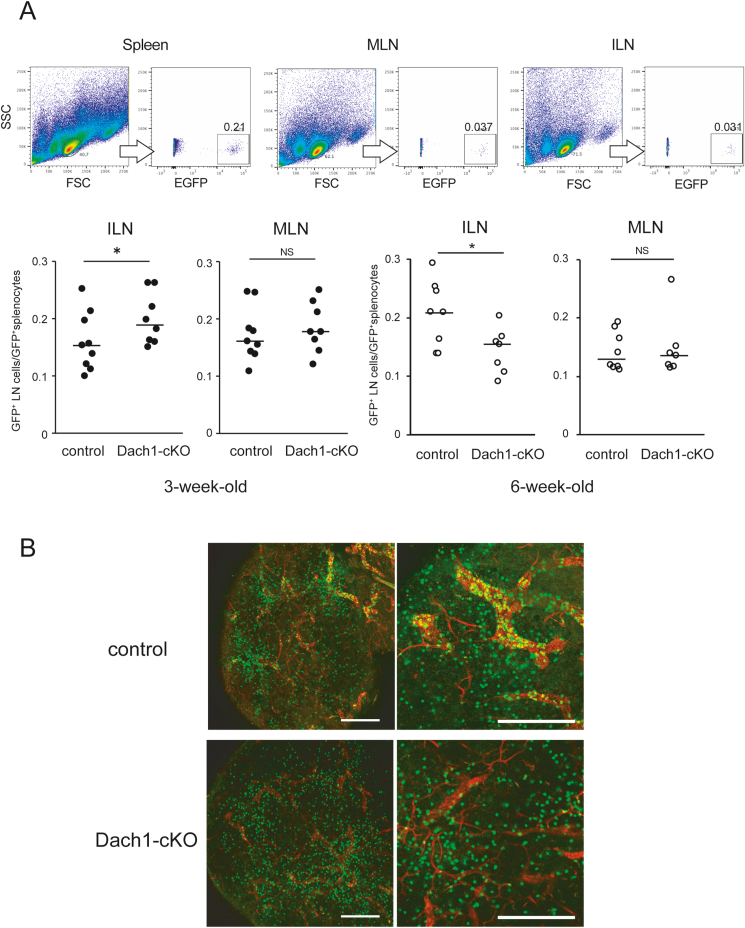
**Short-term lymphocyte migration in Dach1-cKO LNs.** (A) The percentage of GFP^+^ lymphocyte in spleen, ILNs and MLNs of 3-week-old (control; n = 9, Dach1-cKO; n = 8) and 6–8 week-old mice (control; n = 8, Dach1-cKO; n = 7) were analyzed after injection of GFP^+^ cells. Each plot represents the ratio of LN GFP^+^ cells and splenic GFP^+^ cells of the same individual. The Mann-Whitney *U* test was used to analyze the data. *, P < 0.05, NS: not significant. (B) Whole-mount images of ILNs from control and Dach1-cKO mice intravenously injected with GFP^+^ lymphocytes. Representative images of 3 control and Dach1-cKO LNs are shown. Scale bar: 200 μm.

To examine whether the *Dach1* deficiency would affect lymphocyte trafficking into LNs *in vivo*, we performed an adoptive transfer experiment where we analyzed migrated splenocytes from GFP transgenic mice into control and Dach1-cKO LNs. Despite low PNAd expression, lymphocytes migrated more efficiently into 3-week-old Dach1-cKO inguinal LNs than the controls (Fig. 6A). Immunostaining with anti-TER-119 antigen, a marker for erythroid cells, revealed red blood cell leaks in 3-week-old KO ILNs, whereas not in 6-week-old, suggesting that deficiency of DACH1 affects barrier function of blood vessels in 3-week-old ILNs (Fig. S7). In contrast to 3-week-old ILNs, lymphocytes migrated less efficiently into 6-week-old Dach1-cKO LNs than controls, consistent with the lower PNAd expression. Whole mount analysis revealed that the number of vascular-bound GFP^+^ lymphocytes detected after intravenous injection was apparently reduced in the Dach1-cKO LNs compared with control, suggesting that the lower PNAd expression in Dach1-cKO LNs affects lymphocytes binding to HEV-ECs (Fig. 6B). These findings suggest that Dach1 deficiency affects PNAd expression levels and lymphocyte trafficking in LNs, although the effect varies depending on age.

## 4 Discussion

At embryonic stages, mouse *Dach1* is expressed in the hypothalamus, neocortex, limb buds, and genitalia (Backman et al., 2003; Caubit et al., 1999; Davis et al., 1999). However, DACH1's contribution to the development of these tissues remains unknown (Davis et al., 2001). We found that DACH1 is expressed on MAdCAM-1^+^ CD34^+^ (HEV-type) vascular-like structures in neonatal MLNs and ILNs but not on MAdCAM-1^+^ marginal sinus cells in the spleen, suggesting that DACH1 expression is associated with vascular development in LNs. On the other hand, DACH1 is also expressed on MAdCAM-1^-^ CD34^+^ structures in LNs and PPs. Considering a previous single-cell RNA sequencing analysis revealed that there are CD34^+^ non-endothelial stromal cell subsets in lymph nodes (Rodda et al., 2018), DACH1 is possibly expressed not only in ECs but also in stromal cells in LNs and PPs. DACH1 expression has previously been documented in fetal PPs, where it is expressed in lymphoid tissue organizer cells 10 to 30 times higher than in MLNs (Okuda et al., 2007). Further analysis is needed to characterize those DACH1-expressing cell subsets in the secondary lymphoid organs.

We demonstrated that DACH1 expression is detected in LNs from the late embryonic stage to the first day after birth and sharply decreases from the second day after birth, implying that it functions temporarily in HEV development. It has also been reported that DACH1 is temporally expressed at the vascular bifurcation during early arteriogenesis in the heart, where it is involved in angiogenesis by activating chemokine *Cxcl12* expression and endothelial cell migration against blood flow (Chang et al., 2017; Raftrey et al., 2021). The similar transient DACH1 expression pattern between arteries and HEVs suggests a common role of DACH1 in angiogenesis in LNs and the heart.

The conditional *Dach1*-deficient mice in this study exhibited a reduced proportion of CD34^+^ cells in neonatal LNs. A previous report demonstrated that the Tie2-Cre transgenic mice showed pan-endothelial Cre expression (Kisanuki et al., 2001), suggesting that DACH1 in ECs functions in angiogenesis in developing LNs. On the other hand, Tie2 is expressed in hematopoietic stem cells and myeloid lineage cells (Yano et al., 1997). In this study, we cannot formally rule out the possibility of DACH1's contribution to vascular development in hematopoietic lineage cells.

We demonstrated that DACH1 deficiency reduces PNAd expression in peripheral lymph node vessels. In contrast to ILNs, *Dach1* deficiency results in no effect on PNAd expression in adult MLNs, suggesting that the level of DACH1 contribution varies between LN types. MLN may be more exposed to environmental factors after birth than peripheral LNs, eliciting a persistent immune response that leads to DACH-independent HEV development. The findings proposed that there could be an HEV formation mechanism independent of DACH1 expression in mucosal LNs. Given that DACH1 expression is undetectable in adult ILNs, we hypothesize that endogenous DACH1 functions predominantly during the neonatal period, and that the decrease in PNAd expression in adult *Dach1*-deficient ILNs is the result of fetal vascular abnormalities. Alternatively, DACH1 regulates PNAd expression in adult ILNs, though at a much lower level than during the perinatal period. DACH1 functions as either a positive or negative regulator of multiple gene promoters (Chang et al., 2017; Wu et al., 2011; Chen et al., 2015; Xu et al., 2017; Watanabe et al., 2011; Yu et al., 2019). PNAd molecules are formed following a series of posttranslational modifications by several glycosyltransferases (GalT), including β3GalT, Core1-or Core2-β3GLcNAcT, LSST, β4GalT, ST3Gal, and FucT-VII (Weinstein and Storkus, 2016). A genome-wide in silico promoter analysis revealed that a human DACH1 binding sequence is located within 2-kb upstream of the transcription initiation region of β4GalT-3 and β3GalT-2 (Zhou et al., 2010). Thus, this may necessitate further study of the mechanism underling *Dach1* deficiency in PNAd in the future. Identifying additional target genes regulated by DACH1 will lead to a better understanding of the molecular mechanism of HEV-specific gene expression in LNs.

It is unclear whether HEV development in lymphoid tissue is equivalent to HEV-like angiogenesis in chronic inflammation seen in pancreatic islets and rheumatoid arthritis in non-obese diabetic mice (Faveeuw et al., 1994; Michie et al., 1993). Although we were not able to detect DACH1 expression in HEV-like blood vessels ectopically generated in chronically inflamed tissues, *Dach1* is possibly expressed at low but detectable levels in blood ECs as shown in the ImmGen database of adult mouse (Heng et al., 2008). Our immunohistochemical data suggest that DACH1 is unlikely to play a major role in HEV angiogenesis in inflamed LNs, however, they do not provide sufficient evidence to exclude a function of DACH1 in inflammatory conditions. During steady-state LN vascular turnover and immune responses, HEVs are newly formed by branching from pre-existing HEVs that contain capillary ECs with stem cell properties (Mondor et al., 2016). In our previous study, neonatal HEV-type ECs demonstrate immature properties and express various genes found in stem cells (Hayasaka et al., 2010), suggesting a possible role of DACH1 in stem cell properties of endothelial precursors during LN development. Further studies are required on the difference in DACH1-dependency between inflammation and development.

## 5 Conclusion

In this study, we found that the transcription factor DACH1 is more predominantly expressed in developing HEV-type ECs than in non-HEV-type ECs. The proportion of PNAd^+^ cells in peripheral LNs was significantly reduced in vascular-specific *Dach1*-deficient mice, resulting in lower lymphocyte migration into LNs. These findings suggest that DACH1 plays a role in HEV formation and function in peripheral LNs.

## Funding sources

This work is supported by the 10.13039/501100001700Ministry of Education, Culture, Sports, Science and Technology, Japan (Grant-in-Aid for Scientific Research, 19K07278 to H.H.), and an external funding support research grant from Graduating School of Science and Engineering, 10.13039/100012043Kindai University, Japan.

## CRediT authorship contribution statement

**Arisa Shintani:** Investigation. **Shoko Fukai:** Investigation. **Reika Nobusawa:** Investigation, Validation. **Kanako Taniguchi:** Investigation. **Tomohiro Hatatani:** Formal analysis. **Hayato Nagai:** Investigation. **Tomohiro Sakai:** Investigation. **Takuji Yoshimura:** Resources. **Masayuki Miyasaka:** Supervision, Conceptualization. **Haruko Hayasaka:** Project administration, Writing – original draft, Writing – review & editing.

## Declaration of competing interest

The authors declare that they have no known competing financial interests or personal relationships that could have appeared to influence the work reported in this paper.
