# Supplementary material for: Dach1 transcription factor regulates the expression of peripheral node addressin and lymphocyte trafficking in lymph nodes
Source: Curr Res Immunol. 2022 Aug 21;3:175–85. doi: 10.1016/j.crimmu.2022.08.008 (PMC9421177; doi:10.1016/j.crimmu.2022.08.008)
Supplement: Multimedia component 1 [file mmc1.pdf]

Antibody and reagent	Source	Clone or identifier	Concentration (μg/ml)
Rabbit anti-DACH1	ProteinTech Group, Inc.	10914-1-AP	1:200 dilution
Biotinylated rat anti-mouse PNAd	BioLegend	MECA-79	2.5 or 10
Biotinylated rat anti-mouse MAdCAM-1	In-house	MECA-89 or MECA-367	2.5 or 10
eFluor660 rat anti-mouse CD34	Thermo Fisher Scientific	RAM34	1.5 or 2
Alexa Fluor647 rat anti-mouse CD31	BioLegend	390	1
Alexa Fluor488 hamster anti-podoplanin	BioLegend	8.1.1	1
Biotinylated rat anti-mouse TER-119	BioLegend	Ly-76	5
Biotinylated rabbit anti-mouse CCL21	Peprotech	500-P114	1:200 dilution
Biotinylated rat anti-mouse Nepmucin	ZAQ5	Gift from Dr. E. Umemoto, University of Shizuoka	2
Rat anti-Mouse Autotaxin	S9A9	Gift Dr. J. Aoki, Tohoku University	5
FITC rat anti-mouse CD45	TONBO biosciences	30-F11	1
PE/Cy7 rat anti-mouse CD45	BioLegend	30-F11	1
Streptavidin-PE	Invitrogen	12-4317-87	1
Streptavidin-Alexa Fluor488<br>Streptavidin-Alexa Fluor 568<br>Streptavidin-Alexa Fluor647	Thermo Fisher Scientific	S11223, S11226, S21374	2
7-AAD	BioLegend	420404	1
Hoechst 33342	Thermo Fisher Scientific	H1399	3

Table S1. List of antibodies and reagents used in this study.

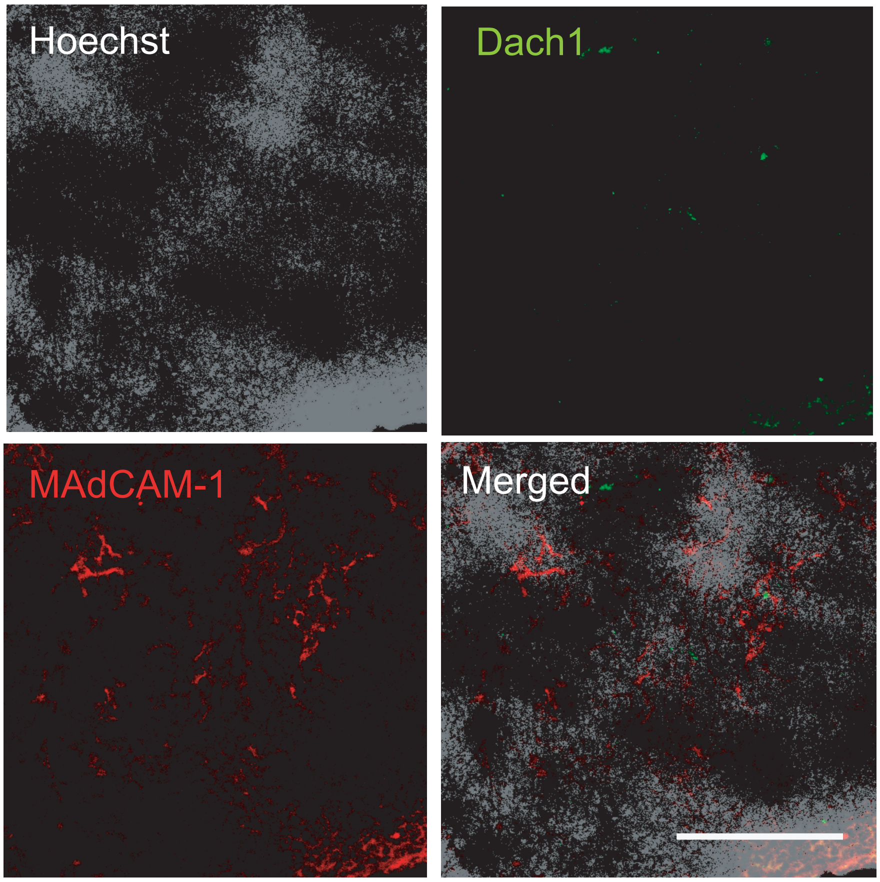

Fig. S1 Detection of DACH1 in newborn spleen. Immunohistochemistry was performed to detect DACH1 (green) and MAdCAM-1 (red). Nuclei were stained with Hoechst (gray) . Data are representative of two experiments with two mice. Scale bar: 100 μm.

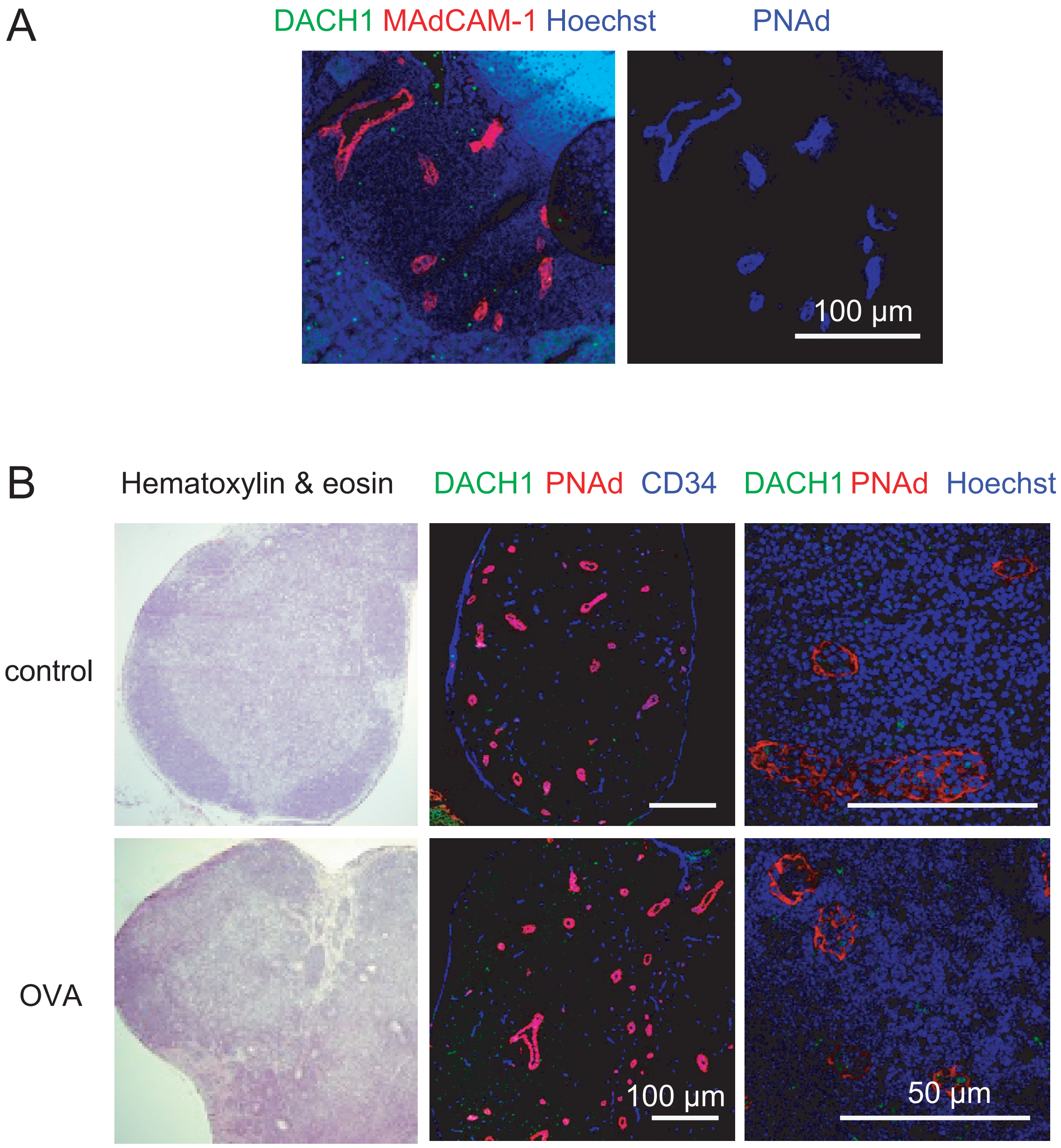

Fig. S2 Dach1 expression in adult tissue chronic and acute inflammation.

(A) pancreas of 13-week-old NOD mice and (B) Axillarly lymph nodes of 8-week-old mouse 4 days after OVA immunization were collected and subjected to (HE) and immunohistochemistry. Data are representative of 3 mice (A) and two experiments with 4 mice in each group (B).

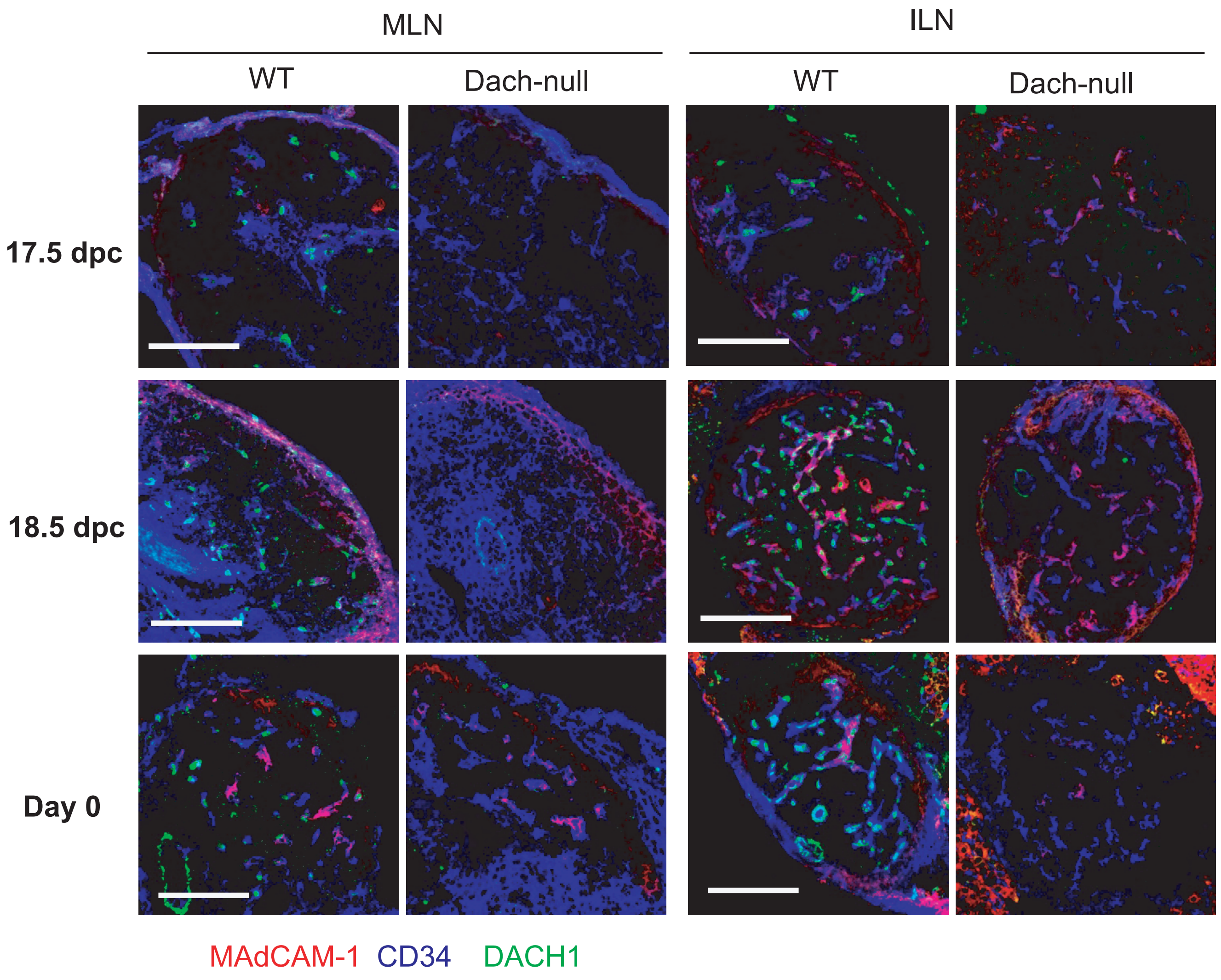

Fig. S3 The DACH1 expression in wild-type and Dach1-null lymph nodes.

MLNs and ILNs were collected from wild-type and Dach null mice, and the expression of DACH1, MAdCAM-1, and CD34 was analyzed by immunohistochemistry. Data are representative of 6 experiments with 6 mice in each group. Scale bar: 100 μm

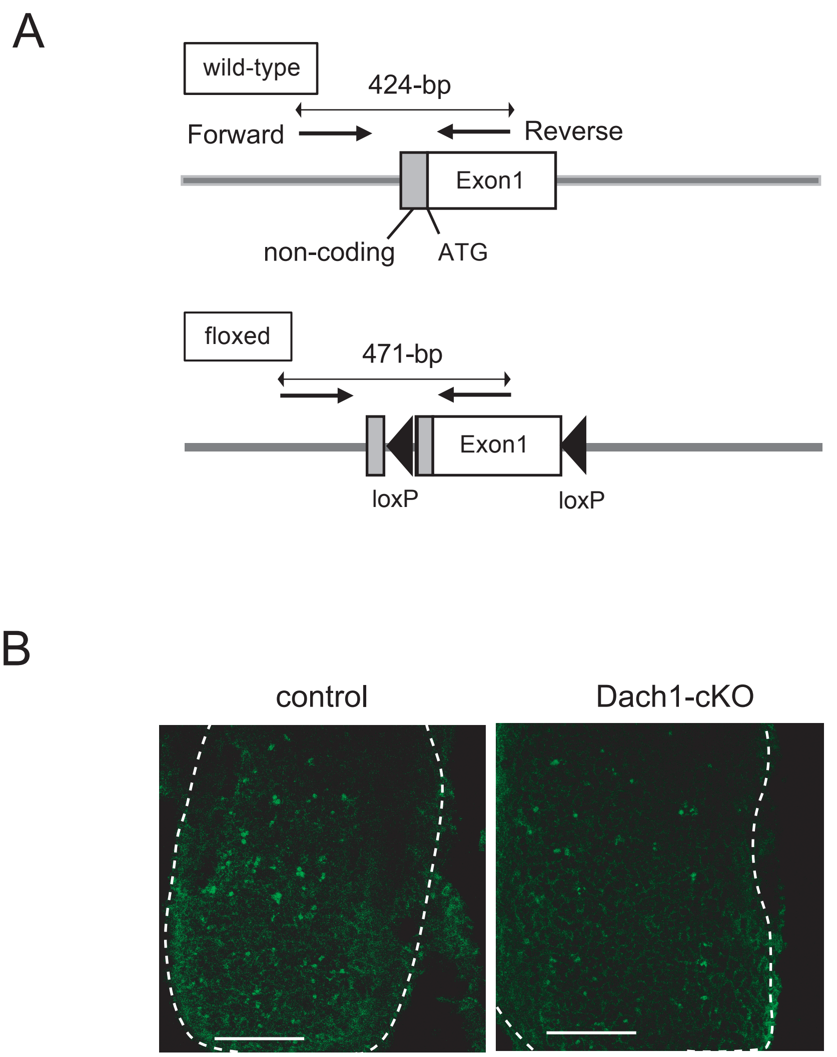

Fig. S4 Generation of vessel endothelium-specific Dach knockout mouse. (A)Schematic representation of the selected Dach1 gene targeting strategy. Gray rectangles show non-conding sequence and ATG represents the initiation codon in Exon1. The binding sites of the screening primers and the size of PCR products are shown by arrows.

(B) DACH1 expression in newborn control and Dach1-cKO MLNs. Representative images with 2 littermate mice in each group are shown. Scale bar: 100 μm.

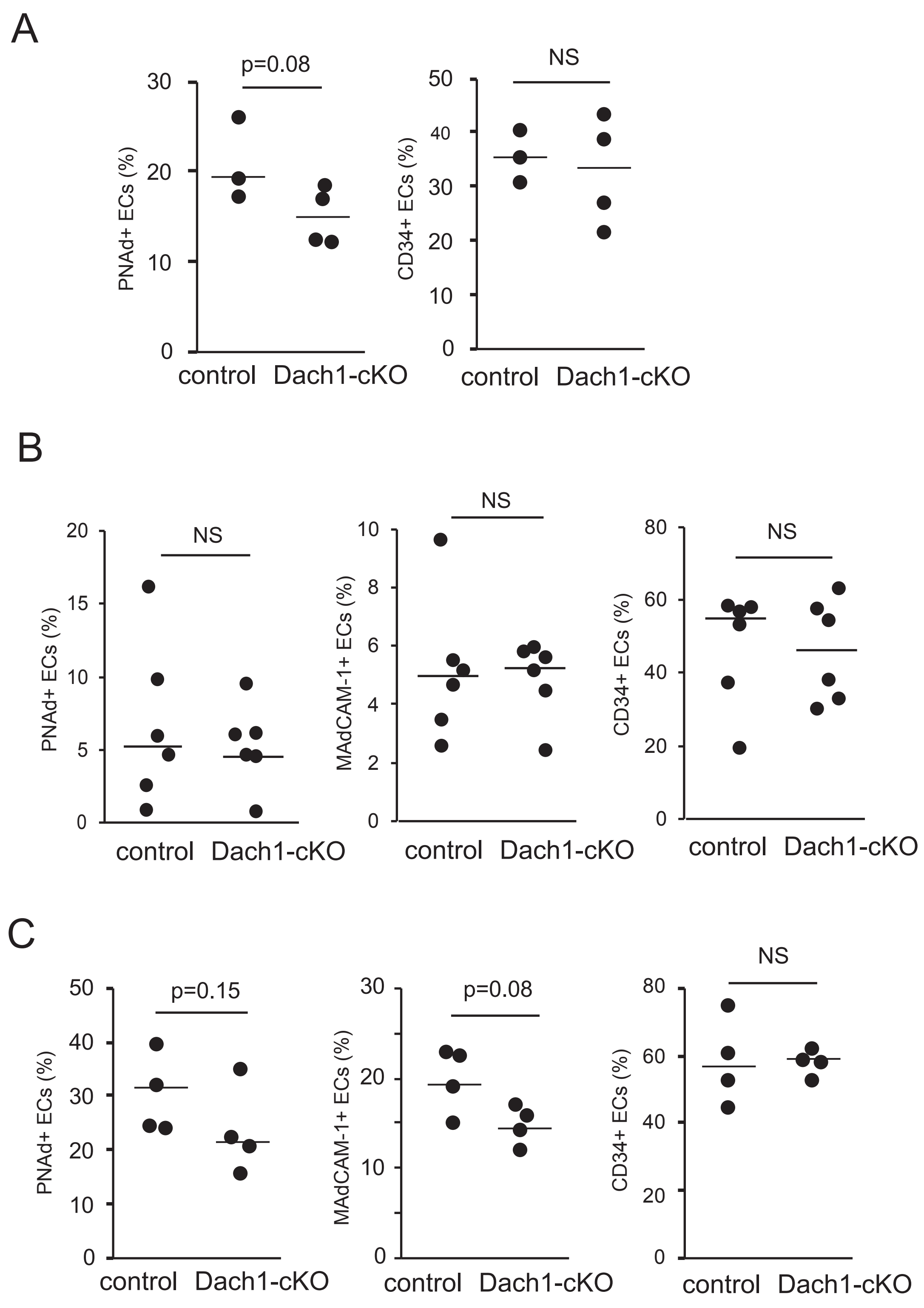

Fig. S5 Flow cytometric analysis of PNAd, MAdCAM-1 and CD34 expression Dach1-cKO lymph nodes. The percentage of $CD34^{+}PNAd^{+}$ or $CD34^{+}MAdCAM\text{-}1^{+}$ cells of $7\text{-}AAD^{-}$ $CD45^{-}$ cell populations are shown. Each plot represents the value of each sample. (A) ILN cells of 6-week-old control (n=3) or littermate Dach1-cKO (n=4) were subjected to analysis. (B) MLN cells of 3-week-old ittermate mice per group were pooled. Each plot shows the value of each pooled sample in 6 experimental repeats. (C) MLN cells of 6-week-old mice (n=4) were subjected to analysis. Mann-Whiteney's U test was used as the significance test. NS: not significant.

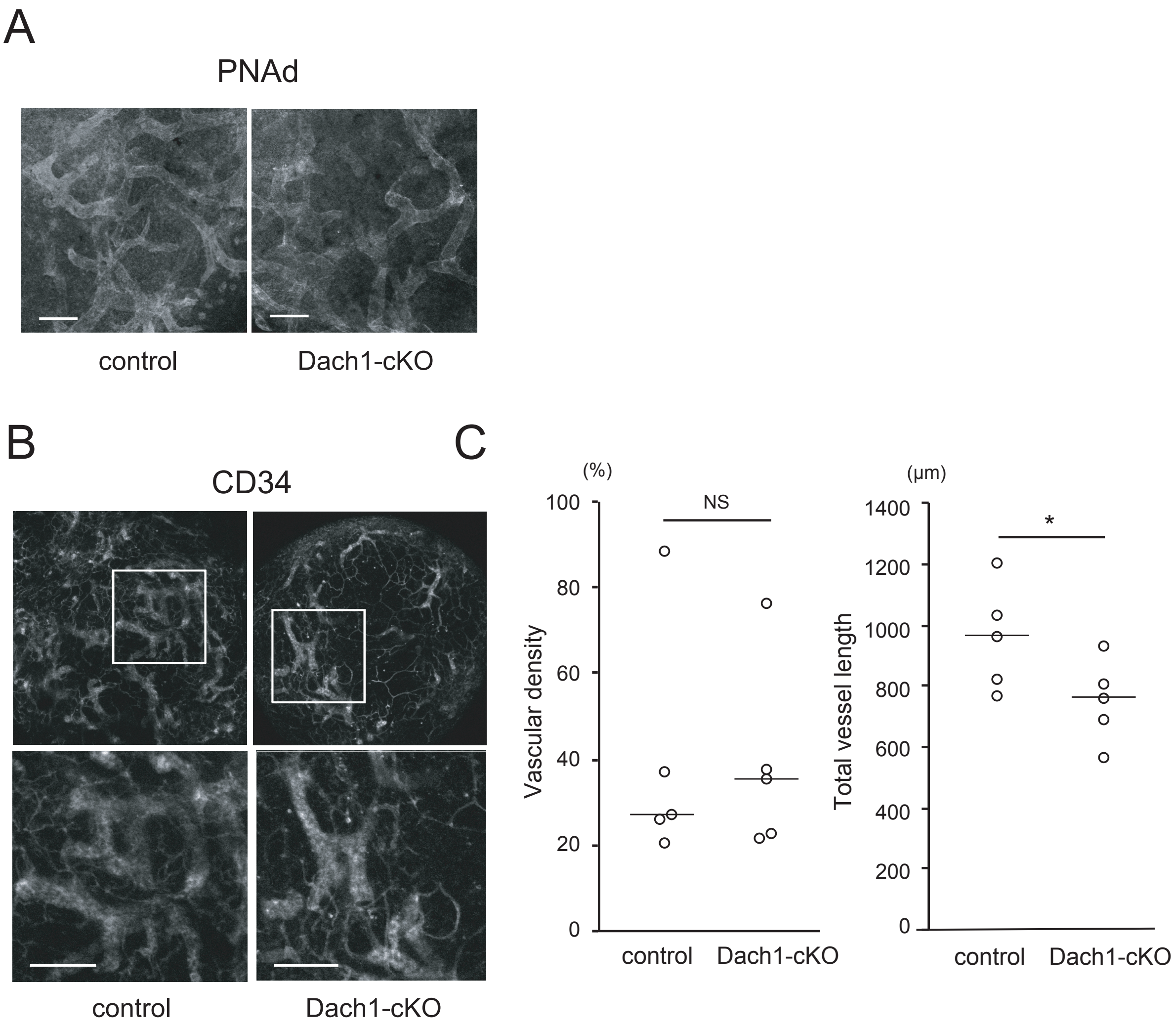

Fig. S6 The vascular architecture of wild-type and Dach1-cKO lymph nodes. (A) PNAd^{+} HEVs in 3-week-old popliteal LNs were detected with biotinylated anti-PNAd mAb and streptavidin-conjugated Alexa Fluor 568. Representative z-projection images of LNs from 5 control or 6 Dach1-cKO mice are shown. (B) CD34^{+} blood vessels in 6-week-old wild-type and Dach1-cKO pLNs were detected by intravenous injection with eFluor 660 anti-CD34 mAb. Representative z-projection images of 5 LNs from 3 control or 3 Dach1-cKO mice and the region of interest (ROIs: approximately 53,000 μm^{2}) covering large blood vessels are shown. (C) Quantitative data of vascular density (CD34^{+} area/ total area * 100%) and total vessel length in ROIs. Mann-Whiteney's U test was used as the significance test. Each plot in the scatter plot represents a single tissue section's value, and each line represents the median. *, $P < 0.05$, NS: not significant. Scale bar: 100 μm.

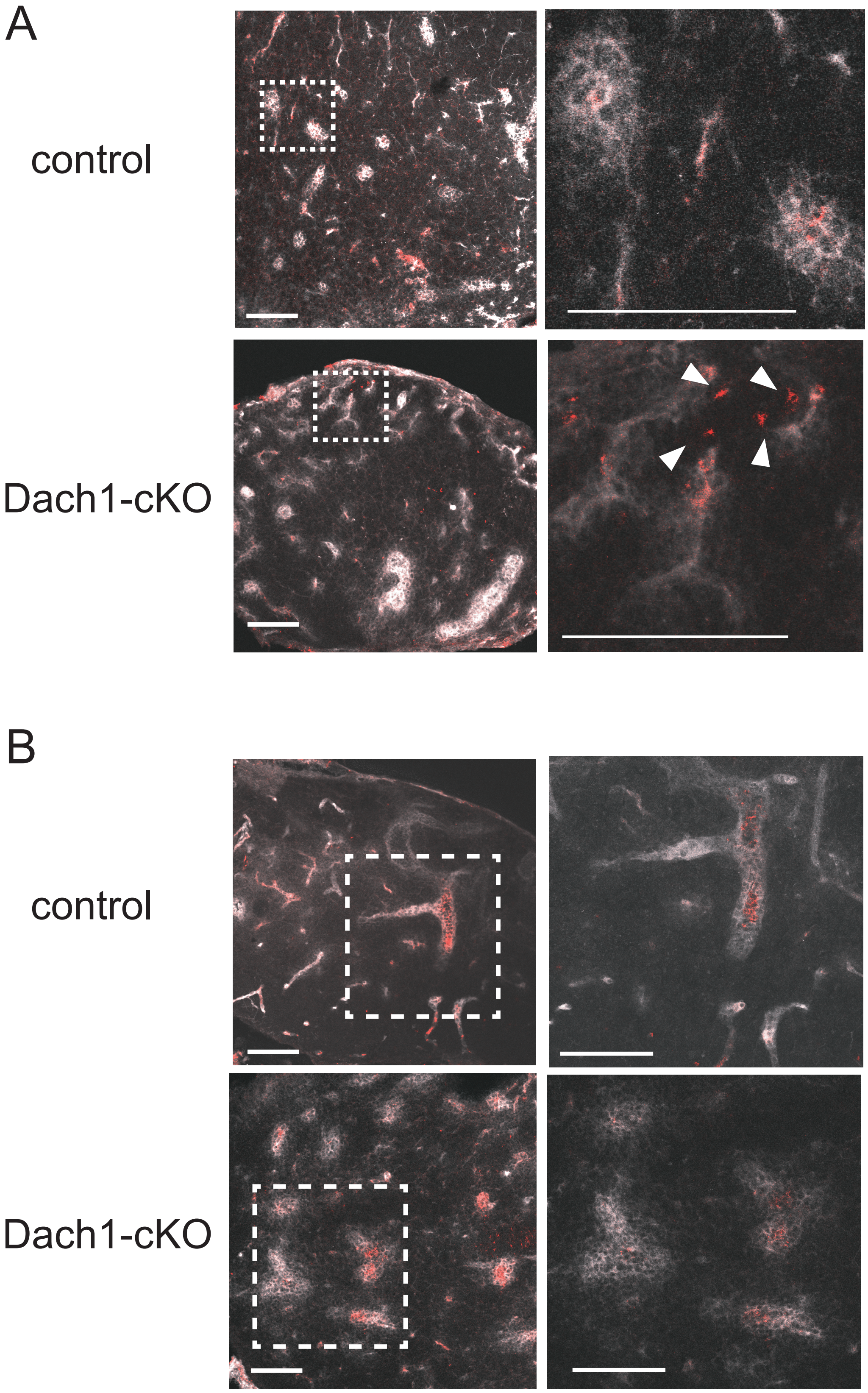

Fig. S7 Comparison of blood vessel permeability of control and Dach1-cKO ILNs. Immunohistochemistry was performed to detect TER-119$^+$ RBC leakages (arrowheads) in ILNs. Data are representative of (A) 3-week old ILNs (n=3) and (B) 6-week old (n=2) littermate ILNs stained with anti-CD34 (gray) and anti-TER-119 (red) mAbs. Scale bar: 100 μm.
